# The high need for trials assessing functional outcome after stroke rather than stroke prevention with GLP-1 agonists and DPP-4 inhibitors

**DOI:** 10.1186/s12933-018-0674-3

**Published:** 2018-02-21

**Authors:** Vladimer Darsalia, Martin Larsson, Thomas Klein, Cesare Patrone

**Affiliations:** 1 Department of Clinical Science and Education, Södersjukhuset, Internal Medicine, Karolinska Institutet, Stockholm, Sweden; 2 Boehringer Ingelheim Pharma GmbH & Co. KG, Biberach, Germany

**Keywords:** Glucagon-like peptide 1 receptor, Dipeptidyl peptidase-4 inhibitors, Stroke, Diabetes

Glucagon-like peptide 1 receptor (GLP-1R) agonists and dipeptidyl peptidase-4 inhibitors (DPP-4i) are approved drugs for the treatment of hyperglycemia in patients with type 2 diabetes (T2D) [1].

In addition to these anti-diabetic properties, in 2009 Li et al. showed that the activation of GLP-1R by exendin-4 could also reduce brain damage in a mouse stroke model [2]. Darsalia et al. in 2013 showed similar results by using the DPP-4i linagliptin [3]. Over the last two decades, more than thirty preclinical studies have confirmed the efficacy of both GLP-1R agonists and DPP4i in different animal models of stroke (with or without T2D) (reviewed by Darsalia et al. [4]). The efficacy in these studies consisted in reduced brain damage and improved functional parameters after acute, chronic or delayed drug administration in experimental stroke.

In the past few years, several large cardiovascular outcome trials employing GLP-1R agonists and DPP-4i in human subjects with T2D at increased cardiovascular risk have been concluded (reviewed by Nauck et al. [5], Avgerinos and Tziomalos [6] and Hemmingsen et al. [7]). For the GLP-1R agonists, some of these studies have shown reduced cardiovascular mortality (LEADER employing liraglutide [8]) and stroke incidence (SUSTAIN-6, employing semaglutide [9]). Others have been neutral; ELIXA employing lixisenatide [5] and the latest addition to these large outcome trials, the EXSCEL study employing the GLP-1 analogue exenatide [10]. For the DPP-4i trials the results so far have been neutral [5, 11]. Recently, a few meta analysis studies using the data from these large clinical studies have been published. The picture remains essentially the same with reduced cardiovascular and all cause mortality but neutral effects on stroke incidence (semaglutide in SUSTAIN-6 being the only GLP-1R agonist with significant effect on stroke incidence) by GLP-1R agonists [12, 13] and neutral results by DPP-4i [12, 14, 15]. The measure of the efficacy in all these trials was stroke incidence and stroke mortality.

In summary, the definition of “efficacy” in preclinical and clinical studies has been fundamentally different, with preclinical studies addressing functional outcome after stroke and clinical studies addressing stroke incidence and death. Despite these essential differences, the overall message to the neuro and diabetic communities has been that: (1) the efficacy of GLP-1R agonists in animal models of stroke has been confirmed in some clinical studies using GLP-1R agonists (2) the efficacy from DPP4i shown in animal studies has been compared with the failure of the large clinical studies to show benefit in stroke prevention. When it comes to DPP-4i, this has led to concerns about the clinical benefit of DPP-4i and questions on why beneficial effects seen in experimental animal models fail to translate into stroke efficacy in the large cardiovascular outcome trials.

This message is not correct. Although the prevention of complications, such as stroke, is a very important target for the management of diabetes, failure to prevent stroke does not indicate the inability to reduce injury and to improve the functional outcome after stroke, which would be essential to reduce the total costs of stroke and patient welfare. Indeed, when comparing clinical outcome studies and preclinical functional outcome studies, it is important to keep in mind that the two trial types are essentially different. The large human long-term treatment trials assessed the prevention of cardiovascular events. In contrast, the experimental trials looked at the modification of the outcome once an event (stroke) has occurred. These are two fundamentally different aspects that cannot be compared and that could explain why DPP-4i have shown positive effects in experimental animal models, but failed to show any benefit to prevent stroke in the large randomized outcome trials so far.

Human small studies addressing whether GLP-1R agonists can modify the outcome of cardiac and neurological conditions do exist. Lønborg et al. demonstrated already in 2012 that the administration of the GLP-1R agonist exenatide to patients with ST-segment elevation myocardial infarction (STEMI) treated with percutaneous coronary intervention improved myocardial salvage index [16]. Importantly, a recent study published on *Lancet* this year and involving 62 patients with Parkinson’s disease (32 treated with exenatide and 30 controls) showed positive effects of exenatide on functional motor scores parameters, which were sustained beyond the period of exposure [17]. This data is in agreement with several functional outcome trials obtained in rodent animal models of Parkinson’s disease in the past fifteen years (reviewed by Athauda et al. [18]) indicating that when animal and human trials evaluate equivalent parameters, translation is possible. Interestingly, CARMELINA^®^ (NCT01897532) and CAROLINA^®^ (NCT01243424) by using the DPP-4i linagliptin, intend to explore post-stroke functional outcome in patients with T2D by using the modified Rankin scale (at day 7/or at hospital discharge as well as at 3–6 months after stroke), and could provide insights to address this gap.

Stroke is a leading cause of major functional disability. T2D both increases stroke incidence and reduces functional stroke recovery [19, 20]. Therefore, when evaluating treatment options it is essential to keep in mind the fundamental distinction between these two aspects. As a consequence, due to the total lack of clinical data addressing the potential efficacy of GLP-1R agonists and DPP-4i on functional parameters after stroke, functional outcome trials using these drugs are highly needed. Preferably both legacy studies, evaluating the functional outcome after the finished outcome trials, and new interventional randomized trials starting within hours after stroke onset.
